# Phosphate of Lime and the Teeth

**Published:** 1884-04

**Authors:** C. M. Wright


### THE DENTAL REGISTER.

VOL. XXXVIII.] APRIL, 1884. [No. 4.

### COMMUNIATIONS.

### Phosphate of Lime and the Teeth.

Read at the Fortieth Annual Meeting of the Mississippi Valley Dental Association by Dr. C. M. WRIGHT.

The dentist who is fond of study, and who can take pleasure in his library, should be glad that he is alive to-day; for he need never have *ennui*. He can, at any spare moment, or during the intervals of dreary labor for daily bread, turn his attention to any of the many fields now open to his mind, and wander therein to his heart's content; finding on every side interesting curiosities and unsolved riddles, the solution of which promises so many benefits to mankind. Even if he cannot, or will not, throw off his coat and take hold boldly and patiently with the enthusiastic workers in any particular field, and is content simply to walk through, looking on at the work as it progresses, his mind will be refreshed by the view; his interest must be unchained, and, by reflex action, his happiness will be increased. If he enters the field marked, "Etiology of Dental Caries," and follows the distinguished and busy laborers (with Miller now at the head, and some noble veterans, who have done splendid work in the past, wiping the sweat from their brows as they, too, rest for a moment to look on with satisfied expectancy at the younger men), he cannot but be deeply interested and benefitted by the knowledge that he will gain of the potent effects of certain acids upon tooth enamel and dentine; of the wonderful parts played by peculiar microscopic fungi upon decalcified tissue; of the possibility of inflammatory changes in the living tissues, and of the curiosities of the minute anatomical structures now more fully revealed than ever before.

If he extends his rambles into the fields of Biology and Embryology, and follows the experiments of the laborers here, in their diligent efforts to unveil the mysteries that envelop protoplasm, and the advances made in the attempts to sharply define the delicate unfoldings and duplications of the embryonic sheets of the germ capsule, after its unaccountable verification from the churning together of feminoid and masculoid elements, he cannot fail to be impressed. If he considers himself a "practical man, he will nevertheless become convinced, after proper meditation, of the possibilities of all this cultivation of these curious fields, in bringing forth crops of real knowledge—"the knowledge which a man can use"—"the knowledge which has life and growth in it, and converts itself into practical power."

If the dentist's tastes do not encourage him to enter these fields, either as a laborer or spectator, he can turn his attention to objects of interest that appear nearer; objects, however, which may never be brought clearly into the focus of our powers until after the gathering in of the crops of these other fields; objects which can, at present, be viewed *mac*roscopically, but which must present new aspects and properties, now hidden, when our knowledge has advanced sufficiently to permit us to prepare them, so to speak, for *mic*roscopic study.

One of these fields, with the gate wide open, is called "*Food in its Relation to Teeth.*"

While we, I repeat, cannot prepare the objects gathered from this field, owing to imperfect knowledge, we can take the grosser physical properties under observation, and make use of our limited knowledge to a limited extent. Let us see:

1. All agree, not only after careful laboratory experiments, but from empirical observations superficially made at the operating chair, that *dense*, structurally perfect, well proportioned tooth tissues, are desirable, not only for the purpose of continuous healthy function, but as the best *barrier* against the attacks of the agents that are continually, and more or less vigorously, operating for their destruction.

2. All agree that structurally perfect tooth tissues, like all other perfect tissues, depend upon the continuous and harmonious satisfaction, one with the others, in past times and present, of the three great systems of circulation in the body, viz.: The Aerial, the Neural, and the Sanguinious.

Admitting, then, that *quality* is of the first importance, and that *quality* is dependent upon harmonious physiological *action*, past and present, can we, at an early or late period in the life history of a tooth, influence its quality by attention to what we to-day believe to be hygenic laws, or by the therapeutic employment of remedies or foods? Laying aside for the present a consideration of the mighty influence, of the great law of inheritance, and the questions that vaguely possess our minds, but upon which we cannot put our fingers, about the incomprehensible facts which lie dormant in the unverified seminal primates, but which will unfold, under certain circumstances, according to some type, which will represent the sum of ages of impressions in the history of the evolution of race—can we by regarding our known, or supposed known, physiological laws, directly prevent pathological conditions of the teeth, or at least directly fortify them against the ever present enemy by an improvement in the quality of their tissues?

Dr. George H. Cushing, in an article entitled, "Pre-Natal Influences as Affecting the Teeth," published in the December No. 1883, of the *Chicago Journal and Examiner*, starts with the proposition that "perfect and healthy children can only spring from perfect and healthy parents," and that we find, "as a general rule no case where either parent is in a perfect physical condition, and often with one or both parents in an absolutely unfit condition to become progenitors, because of their being in a pronouncedly unhealty condition." It is Dr. Cushing's serious belief that notwithstanding this difficulty, strict attention on the part of the mother to hygienic laws, from the time of conception, will influence, for good, the structural quality of the developing tissues of the teeth of the child.

Chemical analysis has shown that phosphate of lime is an important ingredient of tooth tissues, and it is deemed necessary that the requisite quantity of the phosphate of lime should be supplied in the food, and that the diligent following of the supposed laws of health in regard to exercise, rest, ventilation, bathing, and other habits, will enable this phosphate to be assimilated and properly appropriated by that form of protoplasm the particular function of which is to form tooth tissue. Dr. Cushing says that, even when from wilfulness or capriciousness, hygienic measures will not be regarded by the mother, that the habitual use of phosphate preparations, either as Sargent's preparation of wheat phosphate, or as the syrup of lacto-phosphate of lime, during pregnancy and lactation, will "very likely prove beneficial to the teeth of the children;" also, "that in many cases where the phosphates fail to be assimilated from the natural food, they do seem to be from other sources."

He believes that we are justified by experience and observation in attempting "to supply, by artificial means, or by especially prepared foods, the elements which fail to be assimilated in the ordinary way." Just here I should like to say, in reference to the opinions of Dr. Cushing, and of the others whose opinions I shall take the liberty of using, that we dare not despise, careful clinical observations, even if they should prove to be at variance with pathological laboratory observations, or, at least, not substantiated by laboratory research. Both should go, hand in hand, and where they seem at variance a more careful reconsideration of all the observations should be made.

Austin Flint has recently impressed upon the medical world the importance of the clinical study of diseases as a safeguard against errors arising from exclusive attention to histological researches. The question of food, in health and disease, is not a new one; but its importance is increasing as our knowledge of physiology increases.

The value of certain kinds of food in excess, in certain diseases, and the necessity for a particular reduction of the daily amount of certain other kinds of food in certain other diseases, is recognized by medical men. The careful classification of the ingredients of the body, and of the ingredients of foods, is the work of the physiological chemist, and the physician or dentist who can best recognize the *demands* of the tissues of the body, for certain qualities and quantities of food, serves best the interests of mankind.

The dental profession has, for many years, through the influence of individual dentists of positive convictions, urgently recommended the employment of bread which has not been deprived of its phosphates by too much grinding or bolting of the grain. Dr. John Allen, of New York, has long been an apostle in this work.

Prof. Luther Shepard, of Boston, read an article before the Odontological Society of New York, in December, 1875, on "Food in its Relation to the Teeth." In it is presented the history of a case that had been under observation for several years, in which the teeth, which "were very soft," gradually became "hard and dense," so that "instruments cut with difficulty, and gave out the peculiar, sharp, high tone, so familiar to us, as indicative of well-formed dentine." Prof. Shepard had advised the daily use of oat meal as an article of diet, in addition to the regular mixed diet found on all tables. The patient conscientiously adhered to the prescribed diet, consuming "a saucer of oat-meal twice daily." While admitting "that other causes have, doubtless, contributed their quota to the general result," Prof. Shepard insists that these other causes must "occupy a second place in the production of the result, the patient's general health having undergone no improvement, and no special care beyond what other boys can be induced to take of their teeth having been taken in this case.

In the discussion, which followed the reading of this paper, Prof. Flagg, of Philadelphia, introduced a similar case of marked improvement in the quality of the teeth from the use of oat-meal, cracked wheat, and other such preparations, and gave assent to the general proposition that diet can influence the tooth structure in early life.

Prof. Pierce claimed to have seen a marked improvement in the quality of the teeth, affirming that they had "grown more *dense*" from the use of chalk topically applied, *i. e*, "rubbed in between the interstices of the teeth upon the gums before going to bed." Prof. Pierce does not explain whether the result was accomplished by a new process of nutrition, by *direct feeding* to the tissue, and appropriation by the tissue without the necessity for the tiresome routine generally required of food—of passing into the stomach, undergoing uncomfortable metamorphoses, and working its way into the blood, and thence to remote tissues, or not. Still, the observation may be accepted, that teeth *do* become denser without the addition of oat-meal, and from some other and mysterious causes. *

Prof. Hamilton, of the medical profession of New York, who was present at this meeting of the Odontological Society, said that he had in common with other medical men, given some attention to the destruction of the teeth by materials taken into the mouth, but not to the question of the influence of special diet in improving the quality; but was inclined to the belief that it makes but little difference whether we use oatmeal, potatoes, or beef, provided that what we do eat is properly assimilated; claiming "that the human system is not a crucible, or a barometer," saying "we cannot make a general rule, that we can add to the organism a certain amount of the phosphates by putting a certain amount into the mouth. The amount of phosphates to be used by the system will depend upon the digestion."

I have quoted these reports to show that even in this field that appears so near, and so easy to cultivate, there is much more careful work to be done than has yet been given to it, if we would get even a few sound maxims out of it.

Dr. E. C. Kirk has presented some facts of importance on this subject in a paper published in the *Independent Practitioner* for January, 1884. He details observations made upon the teeth of the pupils of the Pennsylvania Institution for the Deaf and Dumb. Dr. Kirk, having had charge of the *mouths* of the three or four hundred children of this institution, and appreciating

*As we have a multitude of observations of this same thing, found anywhere in the floating literature of the dental profession, the question might be asked, How does this happen? Is it possible that the thorough and constant employment of any such antacid, or cleansing, or antiseptic means as are so frequently recommended, such as soap suds, chalk, carbolic acid, etc., etc., may, by preventing fermentation and the formation of destructive acid, or, by acting as parasiticides in the immediate neighborhood of tooth tissue, *induce*, by the *absence* of deleterious acids, a deposit of the mineral elements in tooth tissue from the general circulation, making soft teeth become dense and hard under the soap suds and chalk? Do the soap suds and chalk stimulate the living matter in the dentinal tubes to supply the so-called dentine of repair, as certain things are said to make hens lay eggs?

the opportunity of observing the influence of a fixed and special diet upon the teeth of such a number of pupils, has tried to give reliable data. He has given the diet table, approved March 1, 1882, for breakfast, dinner, and supper, for each day of the week. I shall not take up your time by quoting the table, rather referring you to a more careful study of Dr. Kirk's article, but will only say that, considering that the institution is a charitable one, the bill of fare is excellent, not only as regards liberality in quantity, but variety and selection. A half pint of milk, bread, and butter, are the staple articles for breakfast for every day, while eggs, Indian mush, oatmeal porridge, or grits, are alternately afforded for certain days. For dinner—roasts of mutton and beef, steaks, fish, and soup follow each other, according to the day of the week, with potatoes, beans, beets or turnips, onions, etc., corn starch, tapioca, bread pudding, etc., to make out the different days' fares. For supper—half a pint of milk, bread and butter as regular articles, with either stewed fruit, cottage cheese, molasses, and ginger cakes as the varying articles.

The children coming to this institution are mostly from the poorer classes, and are not generally in well fed conditions. Their teeth present the varieties of indifferent and poor qualities that we find among badly nourished children. Dr. Kirk has observed a decided improvement in the *density* of the teeth in the course of one year after entering the institution. He says in regard to the improved teeth: "They will be found exceedingly hard and dense, making the wear and tear on cutting instruments very great. Excavators and chisels have to be tempered to the point of brittleness to be effective in preparing cavities; otherwise they will not cut the extremely hard structure." This is, he claims, the "result of repeated tests and observations carefully made." And he says: "But the most conclusive evidence which I have met with of the value of their table diet, so far as the nutrition of their teeth is concerned is the unusual number of cases of arrested caries, and the formation of the so-called secondary dentine." It is to be remembered that the grits, oat meal, and Indian meal for breakfast were added to the diet for the express purpose of improving the quality of the teeth. (I do not know whether upon Dr. Kirk's advice or not.)

Now, from what I have here quoted and for other reasons, I think we are safe in believing that the dental profession accept as plausible and possible, the idea that the *density* of tooth tissues can be increased by proper or special nutrition during the period of early development and during childhood, and that an *excess* of the phosphate of lime in the food, or administered in preparations (just as cod-liver oil, for instance, is, in pulmonary diseases; or, iron preparations in ænemic conditions), *may* be as beneficial to the teeth, as it is believed to be to the osseous system at this period. It is also a generally accepted idea as belief in the profession, that teeth may deteriorate in quality, owing to a lack of these phosphates, in the food at any period of life. It is believed that the teeth of females, during gestation and lactation, are deprived of phosphate of lime for the benefit of the bones and teeth of the fœtus or child. There seems to be no question in the minds of the profession on this point, that phosphate of lime is directly abstracted from the tissue of the teeth of the mother to be used in the development of the bones of the growing fœtus. And it is believed generally that the mineral salts leave the teeth in some diseases of middle life, as in nervous affections, and in wasting diseases from decreased nutrition. Can we say, however, that *enamel* is subject to important retrograde and constructive changes within observable limits, dependent upon food or nutrition? Can we say that *enamel* that is firm and resistant to acids this year, may be soft and non-resistant next year, owing to a non-appropriation of phosphate of lime during the year? Can we assert this with scientific accuracy about dentine? Are we scientifically certain that it is the phosphate of lime that is the varying ingredient? Accepting the chemical operation of an improvement in density in children's teeth, from some cause or other, would it not be well to have laboratory observations based on careful chemical analysis and histological examinations? Chemical analysis of a tooth extracted during the time of faulty nutrition, in childhood or in the pregnant female, should show a lack of phosphate of lime, while a tooth from the same patient after improved diet or the administration of phosphate of lime, should show a larger proportion of this ingredient; or an analysis of the different tissues of a *dense* tooth should be made, and the patient then deprived, as far as possible, of phosphates in the food for a time, and an analysis for another tooth should be made. This experiment could be made on dogs or cats. Until we can bring these things into a scale, we are simply guessing. Until we can make satisfactory physiological and chemical experiments, noting accurately the quantity of the phosphate in the ingesta, and the quantity of waste disposed of in the urine, feces, and perspiration, and can satisfactorily account for the disposition by analysis of the dentine and enamel, etc., how can we say positively, even with our stock of chemical knowledge, that the phosphate of lime in grits, or cracked wheat, or in the syrup of lacto-phosphate of lime, actually goes into the tissues of the teeth? Is it not all supposition on our part, or is it not rather simply deduced from the fact that enamel contains say 855 parts in a thousand of phosphate of lime, and dentine about 643 parts in 1,000; that phosphate of lime is the most important (next to water) of the inorganic constituents of the body; that it exists in a solid form in the teeth and bones, united with the animal matter? Why have we not pitched upon water as the varying ingredient for the same reasons? There is no part, to be sure, of the body which does not contain, or does not yield upon incineration, phosphate of lime. It has been *said* that the bones of animals soften if the animals are fed upon food deficient in this ingredient. It has been *said* that fractures of the bones of pregnant women do not readily unite owing to a lack of the phosphates. It has been found that during continuous mental labor the amount of the phosphate of lime is diminished in the urine, from which has been deduced that it has some connection with nervous activity. Dusant claims that it accummulates wherever tissue changes are rapidly taking place. It is believed to have the property of aiding constructive metamorphosis. Prof. Cassidy, in a paper read before the Cincinnati Dental Society a month or two ago, but which I have not seen in print, and, therefore, cannot quote from, suggested the possibility of a relation between the increase of the nervous diseases of to-day (neurasthema, etc.), and the elimination of important nitrogenous elements from our grain food by fine grinding and bolting of the meal. We are informed by those who have studied the development of enamel, dentine, and cement that imperfectly developed enamel continues imperfect; that dentine becomes denser during life by age; that there is a "dentine of repair" occurring at the peripheral ends of the dentinal tubes, nearest to points of irritation, but have we *proofs* that changes in the amount of phosphate of lime take place to and fro, now less, now more, according to the amount taken into the stomach? What might be the proper course for us as practical men to do, if this proved to be the case?

I have a suggestion to make, based on the floating opinions in the profession, the soundness of which I shall leave with you. It is this: Feed your pregnant women with the best preparations of the phosphates; flood them with phosphates during gestation and lactation; flood the children after they appear with phosphates, until by examination with excavators and chisels you find the enamel and dentine as dense as you could desire, then destroy the pulps of all the teeth, to *prevent* deterioration from any possible lack of this element, or from possible impairment of nutrition in after life!! If this suggestion shocks you, I ask, is it not as reasonable as some theories of specific methods of feeding? And have we not as many instances of the saving of teeth by this method—not, indeed, undertaken with this grand (!) idea in view, but accidentally, as can be produced in favor of the feeding process?

Now, having raised as many questions as I can in a paper of this kind, questions, by the way, which I cannot answer, but which may be of importance as showing what a fine field for work this "practical" one offers, I beg to say in conclusion, that personally, I must add that my own empirical (clinical) observations favor the employment of the preparations of the phosphates for *children*. I have tried them for years, in dozens of recorded cases, and must acknowledge that in some way or another, the result has been as satisfactory as the administration of any remedy for any disease. Children have behaved as though under the influence of a regularly administered tonic. Pale children have acquired color in their cheeks. Listless children have waked to new energy. Flabby children have acquired firmer muscles, and dental caries has been checked to a degree noticeable to parents as well as to myself. I have not believed that the phosphates were simply taken up and deposited in the teeth, but could not fail to observe *general* beneficial results, though unable to fully account for the modus operandi. I have administered it as Dr. Cushing recommends, as the syrup of lacto-phosphate of lime, given daily for a month in tablespoonful doses and discontinued for a month, and so on. I do not know but that a bit of breakfast bacon, or a small piece of juicy beefsteak, every morning, if agreeable to the taste of the child, might furnish just the right quantity, or just the right excess of ingredients, necessary to produce all the beneficial effects noticed from the syrup. I do not know, also, whether the eating of grits and oatmeal in *adult* life, if assimilation is good, may not be as injurious, and have in its train diseases as serious as those which follow the excess of nitrogenous substances. Certainly, if it is as active an agent as we are led to believe by our reported observations on the teeth, it must play an important role in other tissues, not so easily observed, and we cannot confine it to comfortable normal deposits in dentine.

